# IR792-MCN@ZIF-8-PD-L1 siRNA drug delivery system enhances photothermal immunotherapy for triple-negative breast cancer under near-infrared laser irradiation

**DOI:** 10.1186/s12951-022-01255-6

**Published:** 2022-03-02

**Authors:** Yongmei Wang, Haibo Wang, Yuhua Song, Meng Lv, Yan Mao, Hongming Song, Yuanyuan Wang, Gang Nie, Xiaoyi Liu, Jian Cui, Xueqing Zou

**Affiliations:** 1 Breast Disease Center, The Affiliated Hospital of Qingdao University, No. 59, Haier Road, Qingdao, 266071 Shandong People’s Republic of China; 2 Department of Anesthesiology, The Affiliated Hospital of Qingdao University, No. 59, Haier Road, Qingdao, 266071 Shandong People’s Republic of China

**Keywords:** Mesoporous carbon nanocomposite, Zeolitic imidazolate frameworks-8, Triple-negative breast cancer, IR792, PD-L1 siRNA, Near-infrared laser irradiation, HSP70, Dendritic cell

## Abstract

**Background:**

Despite extensive investigations on photothermal therapy, the clinical application is restricted due to poor stability, low therapeutic efficacy of photothermal therapy agents and its affinity loss in the multistep synthesis of delivery carriers. To address this, we designed an IR792-MCN@ZIF-8-PD-L1 siRNA (IM@ZP) nanoparticle drug delivery system. IM@ZP was prepared by in situ synthesis and physical adsorption, followed by characterization. Photothermal conversion ability of IM@ZP was assessed by irradiation of near-infrared (NIR) laser, followed by analysis of its effect on 4T1 cell viability, maturation of dendritic cells (DCs) and the secretion of related cytokines in vitro, and the changes of tumor infiltrating T cells and natural killer (NK) cells in vivo. Subcutaneous 4T1 tumor-bearing mouse and lung metastasis models were established to investigate the role of IM@ZP in killing tumor and inhibiting metastasis in vivo.

**Results:**

IM@ZP was uniform nanoparticles of 81.67 nm with the characteristic UV absorption peak of IR792, and could effectively adsorb PD-L1 siRNA. Under the irradiation of 808 nm laser, IM@ZP exhibited excellent photothermal performance. IM@ZP could be efficiently uptaken by 4T1 cells, and had high transfection efficiency of PD-L1 siRNA. Upon NIR laser irradiation, IM@ZP effectively killed 4T1 cells, upregulated HSP70 expression, induced DC maturation and increased secretion of TNF-α and IL-6 in vitro. Moreover, in vivo experimental results revealed that IM@ZP enhanced photothermal immunotherapy as shown by promoted tumor infiltrating CD8 +  and CD4 +  T cells and NK cells, and inhibited tumor growth and lung metastasis.

**Conclusion:**

Together, biocompatible IM@ZP nanoparticles result in high photothermal immunotherapy efficiency and may have a great potential as a delivery system for sustained cancer therapy.

**Graphical Abstract:**

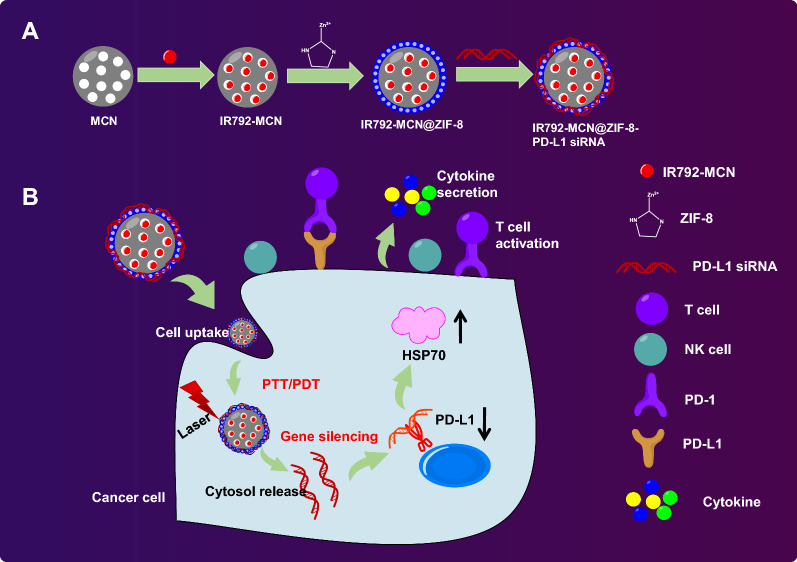

**Supplementary Information:**

The online version contains supplementary material available at 10.1186/s12951-022-01255-6.

## Background

Breast cancer is the most frequently diagnosed cancer in women and a leading cause of premature mortality among women [1]. Triple-negative breast cancer (TNBC) is a heterogenous subtype of breast cancer characterized by lack of estrogen receptor, progesterone receptor and overexpression of human epidermal growth factor receptor 2 gene [2]. TNBC is rather aggressive and commonly presents resistance to chemotherapy [3]. Emerging potential of photothermal immunotherapy has been found to repress tumor growth and enhance immune response of hosts, which, however, is also limited by unfavorable immunosuppression [4]. Hence, an urgent need exists for the identification of effective and safe therapeutic approaches to prevent primary tumor and the resultant metastasis.

Mesoporous carbon nanocomposite (MCN) has been reported to be a viable material for magnetic solid-phase extraction of trace sulfonamides that have been extensively used as antibiotics in clinics, including sulfamerazine, sulfadiazine, sulfapyridine, sulfamethazine, sulfamethoxypridazine and sulfadimethoxine [5]. MCN can not only act as a drug carrier with a high drug loading efficiency but also a photothermal agent for photothermal therapy [6, 7]. Zeolitic imidazolate frameworks-8 (ZIF-8) doped MCN has been identified to exhibit an efficient antibacterial activity [8]. ZIF-8 is a porous carrier with unique features including high loading and pH-sensitive degradation [9], and possesses remarkable antibacterial activity that combines photothermal heating with enhanced antibiotic delivery [10]. In addition, IR792 represents a near-infrared (NIR) laser dye showing great NIR absorption capacity [11]. Nanotherapeutics face some challenges in cancer therapy, such as poor penetration and inefficient accumulation in tumor site; however, under the irradiation of NIR laser, the nanoparticles are thermoresponsive and accumulate in tumor and effectively enter cells, thus stimulating cell apoptosis by damaging mitochondria membrane [12].

In combination with the blockade of programmed death ligand 1 (PD-L1) antibody, a nanodrug carry (Fe_3_O_4_-R837 SP)-involved photothermal therapy under NIR laser irradiation can not only kill the primary tumor but also inhibit the metastatic characteristic of tumor to the lung or the liver [13]. Meanwhile, PD-L1 expression has been observed to be upregulated in MC-38, A20, and 4T1 tumor-bearing mouse models while its blockade contributes to the increased effectiveness of cancer immunotherapy [14]. In light of these reports, we suggested a nano drug delivery system of IR792-MCN@ZIF-8-PD-L1 siRNA (IM@ZP) through in situ synthesis and physical adsorption, and analyzed its promoting effect on the efficacy of NIR-induced photothermal therapy.

## Results

### Fabrication and characterization of IM@ZP

**Fig. 1:**
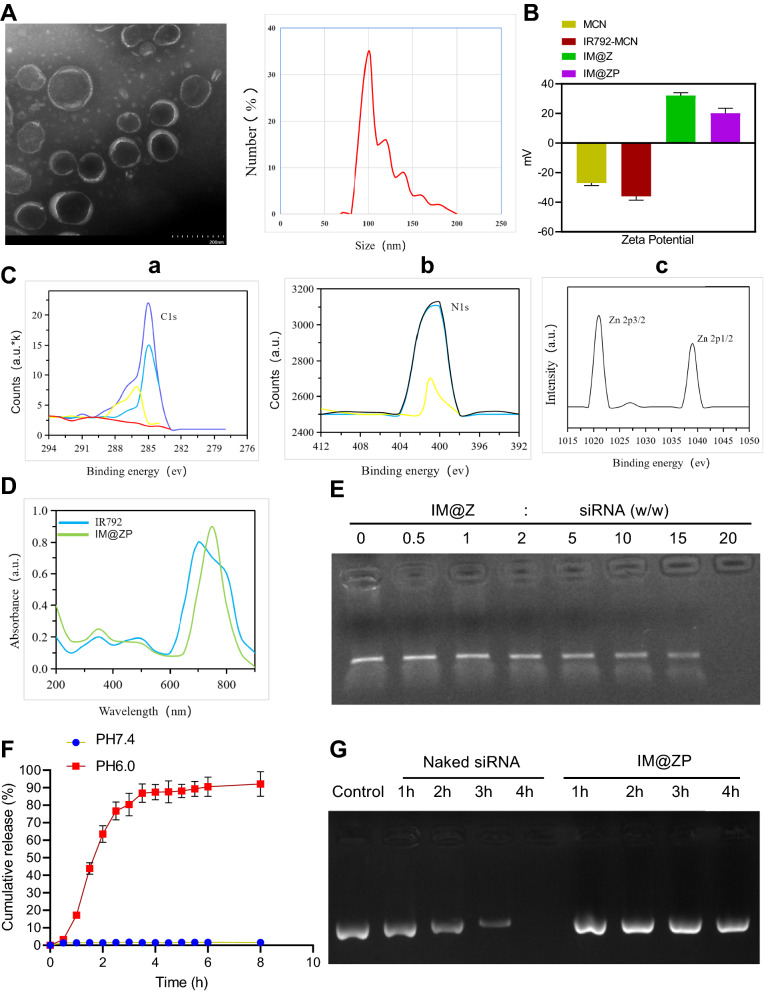
Identification and characterization of the IM@ZP. **A** Representative microscopic views of MCN@ZIF-8 under a TEM and DLS analysis for particle size. **B** The zeta potential of MCN, IR792-MCN, IM@Z and IM@Z -PD-L1 siRNA. **C** XPS spectrums of IM@Z: C1s spectrum (**a**), N1s spectrum (**b**) and Zn 2p spectrum (**c**). **D** UV/VIS absorption spectrum of IR792 and IM@ZP. **E** Gel electrophoresis of PD-L1 siRNA with different mass ratios of IM@Z. **F** Release curves of siRNA when IM@ZP was incubated with PBS at different pH values. **G** Stability of PD-L1 siRNA and IM@ZP in serum evaluated by gel electrophoresis

MCN and MCN@ZIF-8 were subjected to transmission electron microscope (TEM) detection to verify the in situ synthesis of ZIF-8 on MCN, and the mechanism of the formation of silica nanoparticles by hydrolysis of tetraethylorthosilicate (TEOS) was displayed in the Additional file 1: Fig. S1. The results of TEM and dynamic light scattering (DLS) are shown in Fig. 1A, where MCN@ZIF-8 nanoparticles were well dispersed, and each was in a typical spherical structure with the surface covered by film; the average particle size was 82.29 nm, as detected by DLS. In addition, the zeta potential of IR792-MCN changed from negative (− 36 mV) to positive (32 mV) upon ZIF-8 coating (Fig. 1B), which was mainly due to the positive charge of Zn ions in ZIF-8. These results demonstrated the successful synthesis of ZIF-8 film on the surface of MCN.

Then to investigate the surface composition of IR792-MCN@ZIF-8 (IM@Z), we performed X-ray photoelectron spectroscopy (XPS). According to the results (Fig. 1C), the XPS C1s peak fitting of IM@Z was observed (Fig. 1C-a), wherein the strong peak near 284.6 eV was attributed to the sp2 hybrid carbon in the molecular structure of graphite, indicating that the carbon in MCN mainly existed in graphite as conjugate bonds. Meanwhile, there were two peaks near 286.8 and 289.4 eV with relatively low intensity, indicating two types of carbon atoms, N-sp2 C and N-sp3 C, respectively. The explanation for the results was that carbon atoms was replaced by nitrogen atoms [15, 16]. The XPS N1s peak fitting of IM@Z was also observed (Fig. 1C-b), wherein the main peak near 399.6 eV was attributed to the existing form of pyrrole nitrogen, and the small peak near 398.4 eV was about the existing form of pyridine nitrogen [17]. Moreover, in the XPS Zn 2p peak fitting of IM@Z (Fig. 1C-c), peaks at 1021.8 and 1044.5 eV corresponded to the Zn characteristics of ZIF-8, including tetrahedral coordinated Zn and Zinc ion of low coordination number [18, 19].

Further, the UV/VIS absorption spectrum (Fig. 1D) displayed a wide characteristic absorption peak of IM@Z in 600–850 nm, which was similar to that of IR792. This preliminarily demonstrated the successful synthesis of IM@Z. The absorption peak of IM@Z shifted to red at 808 nm, which may be attributed to the aggregation of IR792 in MCN. According to the UV/VIS absorption spectrum, the loading efficiency of IR792 by MCN@ZIF-8 was about 58.94% and the loading capacity was about 0.35 mg/mg. Additionally, Additional file 2: Fig. S2A showed that MCN had strong absorption at 808 nm laser irradiation and could absorb NIR. The photothermal conversion efficiency of MCN, IR792-MCN and MCN@ZIF-8-PD-L1 was subsequently examined under NIR irradiation, as shown in Additional file 1: Fig. S2B. MCN, IR792-MCN, and MCN@ZIF-8-PD-L1 all had certain photothermal conversion under NIR laser irradiation, suggesting that the synthetic IM@Z also has the potential as an enhanced photothermal reagent.

Next, we studied the loading of PD-L1 siRNA on the surface of IM@Z. PD-L1 siRNA and IM@Z were dispersed in DEPC solution treated with diethyl pyrocarbonate, and following 1 h of incubation, the zeta potential of IM@Z changed from 32 to 20 mV (Fig. 1B), indicating that the siRNA with negative charge was adsorbed on the surface. As illustrated in Fig. 1E, IM@Z had obvious fluorescence compared to pure siRNA. On the contrary, with the increase of the mass ratio, the fluorescence exhibited a progressive decrease, which indicated that more siRNA was adsorbed by IM@Z, resulting in the decrease of the remaining siRNA in the supernatant after centrifugation. When the mass ratio exceeded 15, there was almost no fluorescence of siRNA bands, suggesting that all the siRNAs were adsorbed by IM@Z.

In order to achieve effective gene delivery, the release behavior of siRNA was further analyzed using a fluorescence spectrophotometer. As shown in Fig. 1F, upon IM@ZP incubation with phosphate-buffered saline (PBS) at pH 6.0, the release curve of siRNA showed a rapid release of siRNA in the first 3 h, and then the release got gradually stable until 8 h. The release rate of siRNA was about 92.15%, mainly due to the rapid decomposition of ZIF-8 film under acidic conditions. In contrast, no significant siRNA release was observed when IM@ZP was incubated with PBS at pH 7.4 for 8 h. These results demonstrated that IM@ZP may be pH responsive and can release siRNA rapidly and effectively in an acidic environment.

Meanwhile, gel electrophoresis experimental data presented that the fluorescent band of siRNA was still visible following IM@ZP incubation in serum for 4 h, but the pure siRNA was completely degraded. IM@Z due to steric hindrance protected siRNA from RNase degradation (Fig. 1G). This characteristic is very attractive, because the degradation of siRNA in serum is among the causes of gene therapy ineffectiveness. The aforementioned results indicate a successful construction of IM@ZP with a highly stable and attainable pH response.

### IM@ZP shows efficient photothermal performance in vitro

**Fig. 2:**
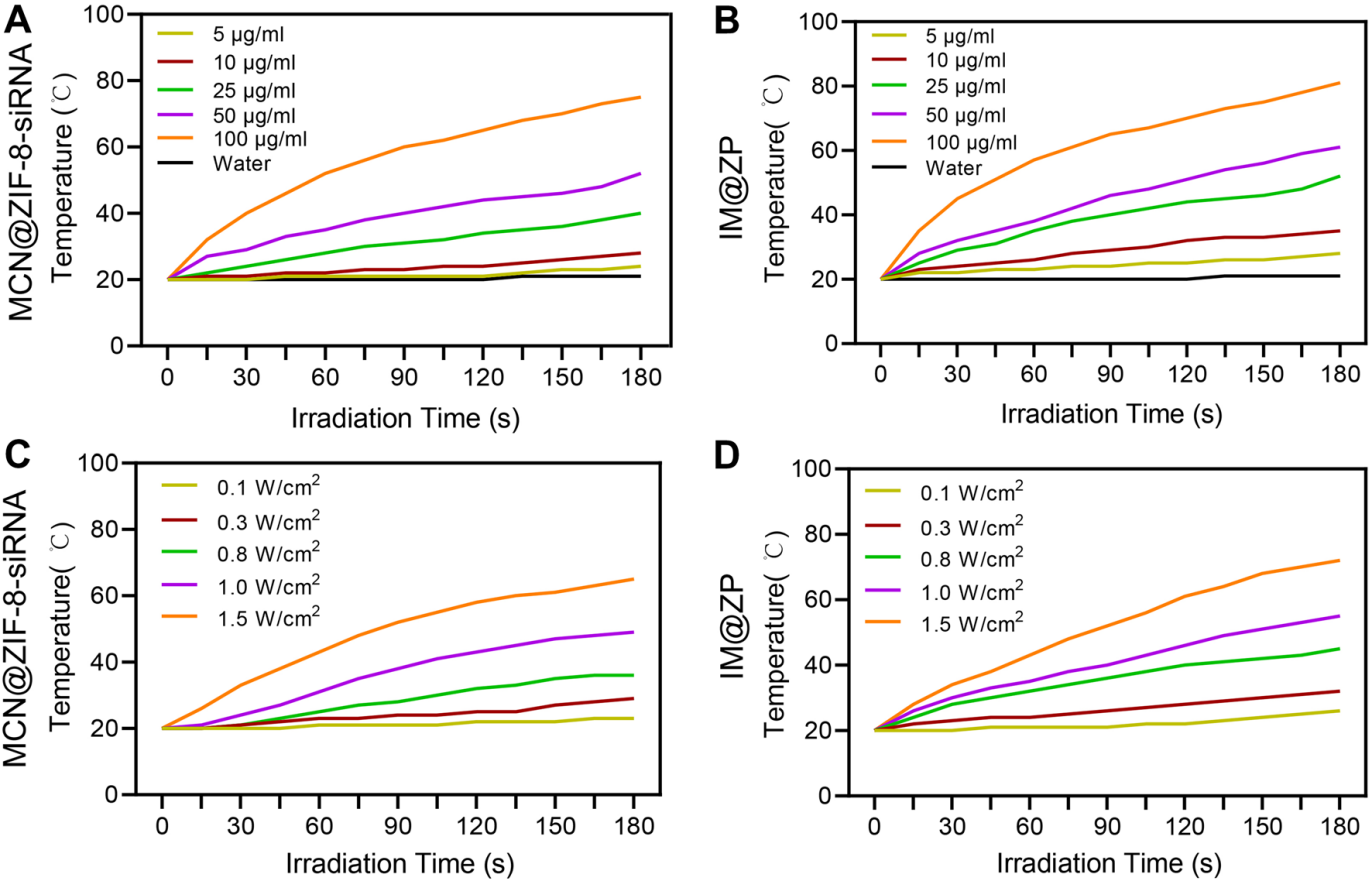
Photothermal effects of IM@ZP. **A** Temperature curves of MCN@ZIF-8-PD-L1 siRNA at different concentrations under 1 W/cm^2^ NIR laser irradiation at 808 nm. **B** Temperature curves of the IM@ZP at different concentrations under 1 W/cm^2^ NIR laser irradiation at 808 nm. **C** Temperature curves of the MCN@ZIF-8-PD-L1 siRNA (50 μg/mL) under NIR laser irradiation at 808 nm at different power densities. **D** Temperature curves of the IM@ZP (50 μg/mL) under NIR laser irradiation at different power densities

Based on the strong NIR absorption of MCN and IR792 at 808 nm laser irradiation (Fig. 1D), we assessed the photothermal effect of IM@ZP. The recorded data of the thermocouple probe revealed that, under NIR laser irradiation of 1 W/cm^2^, the temperature of MCN@ZIF-8-PD-L1 siRNA and IM@ZP increased in a concentration-dependent manner (5–100 μg/mL) (Fig. 2A, B). In addition, under NIR laser irradiation from 0.5 to 1.5 W/cm^2^, the temperature of MCN@ZIF-8-PD-L1 siRNA and IM@ZP (50 μg/mL) was gradually increased (Fig. 2C, D). Importantly, compared with the same concentration of MCN@ZIF-8-PD-L1 siRNA, IM@ZP exhibited an obviously stronger photothermal effect under the same power density of NIR laser irradiation (Fig. 2A, D), which was ascribed to the combination of two photothermal molecules MCN and IR792. Taken together, these lines of evidence indicate that IM@ZP may have excellent in vitro photothermal effects.

### Efficient cellular uptake and transfection efficiency of IM@ZP in vitro

**Fig. 3:**
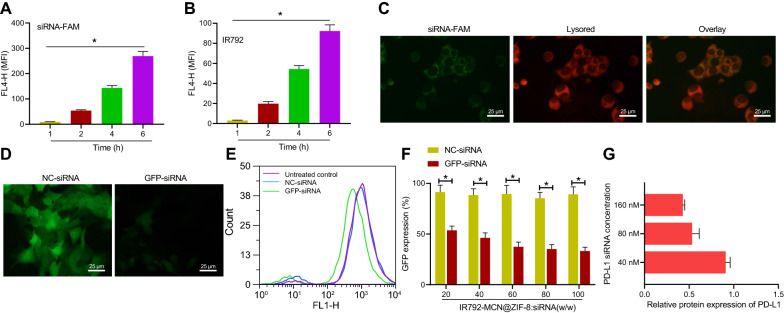
IM@ZP uptake and transfection efficiency in vitro. **A** Quantitative analysis of fluorescence signal intensity of siRNA-FAM in 4T1 cells incubated with IM@ZP for 1, 2, 4 and 6 h analyzed by flow cytometer. **B** Quantitative analysis of fluorescence signal intensity of IR792 in 4T1 cells incubated with IM@ZP for 1, 2, 4 and 6 h analyzed by flow cytometer. **C** CLSM observation of the co-localization of siRNA-FAM (green) and lysosome (red) in 4T1 cells after co-incubation of IM@ZP with 4T1 cells. **D** CLSM images of 4T1-GFP cells incubated with IM@Z and GFP-siRNA at a 80:1 mass ratio (concentration of siRNA was 80 nM), with NC-siRNA serving as the control. **E** Flow cytometric analysis of GFP fluorescence curve in 4T1-GFP cells incubated with IM@Z and GFP-siRNA at a mass ratio of 80:1. **F** Flow cytometric analysis of GFP expression rate in 4T1-GFP cells incubated with IM@Z and GFP-siRNA at different mass ratios. **G** Western blot analysis of PD-L1 protein in 4T1 cells incubated with IM@Z binding to 40, 80 or 160 nM of PD-L1 siRNA, with NC-siRNA serving as the control. *p  < 0.05

Confocal laser scanning microscopy (CLSM) results showed that IR792 (red fluorescence) successfully entered the 4T1 cells (Additional file 3: Fig. S3), showing a high cellular uptake capacity. Moreover, Fig. 3A, B presents that with the increase of incubation time, the fluorescence intensity of siRNA-FAM and IR792 in cells was gradually increased, further indicating that the IM@ZP can be effectively internalized by 4T1 cells. This may be attributed to the existence of ZIF-8 film with positive charge that promotes the binding between the IM@ZP and cell membrane with negative charge.

As shown in Fig. 3C, the MCN@ZIF-8 loaded with siRNA-FAM could be visible in the whole cytoplasm, and part of its fluorescence exhibited co-localization with the LysoTracker Red fluorescence, indicating that the IM@ZP can effectively release siRNA-FAM into the cytoplasm of 4T1 cells. This could be attributed to the uptake of ZIF-8 into lysosomes by 4T1 cells, which degraded in the lysosomal acidic environment, resulting in release of Zn^2+^ to induce reverse ion influx to rupture the lysosomal membrane [20].

In order to achieve the optimal transfection efficiency, CLSM and flow cytometer were used to elucidate the silencing effect of IM@Z at different mass ratios binding to PD-L1 siRNA on the expression of GFP. As compared with NC-siRNA, almost no obvious GFP fluorescence was noted in 4T1-GFP cells when the mass ratio of IM@Z to GFP-siRNA was 80:1 (Fig. 3D). Furthermore, quantitative analysis using flow cytometer demonstrated that the expression of GFP was inhibited in the presence of 80:1 mass ratio (Fig. 3E, F). When the mass ratio of IM@Z to GFP-siRNA was gradually increased, the expression of GFP was gradually decreased in 4T1-GFP cells. GFP expression in 4T1-GFP cells was decreased to the lowest at a mass ratio of 80:1. However, GFP expression was not significantly changed after binding of IM@Z to NC-siRNA at different mass ratios. Therefore, the IM@Z and GFP-siRNA at the mass ratio of 80:1 were used for the subsequent experiments, and the concentration of the loaded siRNA was about 15 nM when IM@Z was 100 μg/mL. Next, Western blot analysis was performed to determine the silencing effect of IM@Z with PD-L1 siRNA on PD-L1 expression, with NC-siRNA regarded as a parallel control. Data depicted in Fig. 3G displayed that IM@ZP significantly inhibited the expression of PD-L1 in 4T1 cells. As the concentration of PD-L1 siRNA in IM@ZP gradually increased, PD-L1 expression gradually decreased in 4T1 cells.

The above results indicate that IM@ZP can be efficiently uptaken by 4T1 cells, thus inhibiting PD-L1 expression.

### Killing of 4T1 cells by IM@ZP under NIR laser irradiation in vitro

**Fig. 4:**
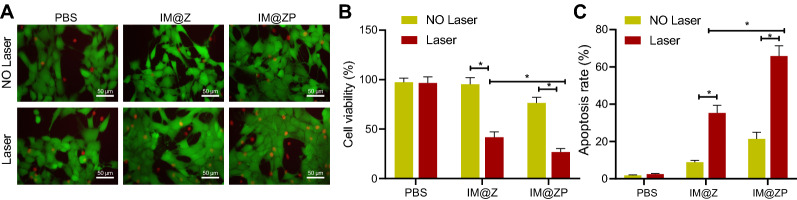
Efficiency of IM@ZP under NIR laser irradiation in killing breast cancer cells in vitro. **A** Calcein AM/PI staining images of 4T1 cells incubated with PBS, IM@Z and IM@ZP with or without NIR laser irradiation (10 min, 1 W/cm^2^) for 12 h (green fluorescence indicates living cells, and red fluorescence indicates dead cells). **B** Viability of 4T1 cells incubated with PBS, IM@Z and IM@ZP with or without NIR laser irradiation (10 min, 1 W/cm^2^) for 12 h measured by CCK-8 assay. *p  < 0.05. **C** Flow cytometric analysis of cell apoptosis following incubation with PBS, IM@Z and IM@ZP with or without NIR laser irradiation (10 min, 1 W/cm^2^) for 12 h. *p  < 0.05

In light of the excellent photothermal effect and gene silencing effect of IM@ZP, we used Calcein AM/PI, CCK-8 and flow cytometric assays to characterize its killing ability on 4T1 cells. The results of Calcein AM/PI staining demonstrated that the number of dead cells was significantly increased in 4T1 cells under NIR laser irradiation as compared with PBS. This suggested that IM@Z had good photothermal killing ability following NIR laser irradiation, and meanwhile, a more pronounced effect was noted following combination with PD-L1 siRNA (Fig. 4A).

In addition, the experimental results of CCK-8 assay showed no significant changes in cell viability following IM@Z treatment (Fig. 4B), suggesting the good biocompatibility of IM@Z. However, cell viability was decreased slightly upon treatment with IM@ZP as compared with IM@Z, which indicated that PD-L1 siRNA alone had little effect on the viability of tumor cells. Notably, when 4T1 cells in the IM@Z and IM@ZP groups were irradiated by NIR laser, cell viability was decreased markedly and IM@ZP led to a more pronounced decrease, which suggested that IM@ZP combined with PD-L1 siRNA could effectively kill 4T1 cells.

Flow cytometric data illustrated that IM@ZP diminished the expression of PD-L1 in the absence of NIR laser irradiation, resulting in a slightly higher apoptosis of 4T1 cells than that of IM@Z. Compared with the IM@Z  +  NIR group, the apoptosis of cells was augmented in the IM@ZP  +  NIR group (Fig. 4C), which indicated that PD-L1 gene silencing could further improve the photothermal killing ability. These results were consistent with those of Calcein AM/PI staining and CCK-8 assay.

Therefore, IM@ZP could effectively kill breast cancer cells under NIR laser irradiation.

### Immune stimulation of IM@ZP under NIR laser irradiation in vitro

We then moved to explore the mechanism of tumor immunity. Western blot analysis indicated an increase of endogenous expression of heat shock protein 70 (HSP70) by 2.3, 2.4 and 2.9 times, respectively in 4T1 cells treated with IM@Z  +  NIR, IM@ZP and IM@ZP  +  NIR (Additional file 4: Fig. S4). This suggested the ability of both PTT and PD-L1 gene silencing to upregulate the HSP70 expression.

**Fig. 5:**
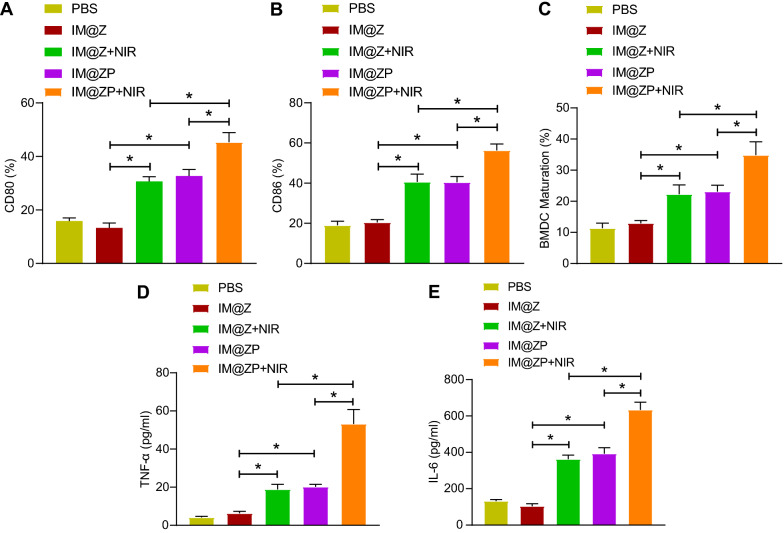
Promoted immune response by IM@ZP under NIR laser irradiation in vitro. **A** Flow cytometric analysis of CD80 expression in DCs cells co-incubated with 4T1 cells treated with IM@Z, IM@Z  +  NIR, IM@ZP and IM@ZP  +  NIR. **B** Flow cytometric analysis of CD86 expression in DCs cells co-incubated with 4T1 cells treated with IM@Z, IM@Z  +  NIR, IM@ZP and IM@ZP  +  NIR. **C** Quantitative analysis of panel **A** and **B**. **D** TNF-α levels in the supernatant of DCs co-incubated with 4T1 cells treated with IM@Z, IM@Z  +  NIR, IM@ZP and IM@ZP  +  NIR measured by ELISA. **E** IL-6 levels in the supernatant of DCs co-incubated with 4T1 cells treated with IM@Z, IM@Z  +  NIR, IM@ZP and IM@ZP  +  NIR measured by ELISA. *p  < 0.05

Additionally, flow cytometric analysis revealed that the maturation rate of DCs was 32% in the IM@ZP  +  NIR group, which was the highest among all groups. On the contrary, in the IM@ZP group without NIR laser, the maturation rate of DCs was 22%. The maturation rate of DCs in the IM@Z  +  NIR group was about 3 times higher than the PBS group (Fig. 5A–C), indicating that PTT can indeed trigger the immune response. Compared to the IM@Z and IM@Z  +  NIR groups respectively, the maturation rate of DCs was also increased in the IM@ZP and IM@ZP  +  NIR groups, indicating that PD-L1 silencing also promoted the maturation of DCs. Furthermore, the highest maturation rate of DCs was noted in the IM@ZP  +  NIR group.

Meanwhile, TNF-α and IL-6 levels were increased in the supernatants of DCs in the IM@Z  +  NIR and IM@ZP  +  NIR groups, as compared with the IM@Z and IM@ZP groups respectively, indicating that PTT could elevate TNF-α and IL-6 levels. Besides, increased TNF-α and IL-6 levels were also noted in the supernatants of DCs in the IM@ZP and IM@ZP  +  NIR groups relative to IM@Z and IM@Z  +  NIR groups, suggesting that PD-L1 silencing also promoted TNF-α and IL-6 levels. Furthermore, the highest levels of TNF-α and IL-6 of DC supernatant was noted in the IM@ZP  +  NIR group (Fig. 5D, E).

These results indicate that PTT and PD-L1 silencing can induce DC maturation and promote immune response. Importantly, siRNA-mediated PD-L1 gene silencing can further enhance the photothermal immune effect.

### Immune stimulation of IM@ZP under NIR laser irradiation in vivo

**Fig. 6:**
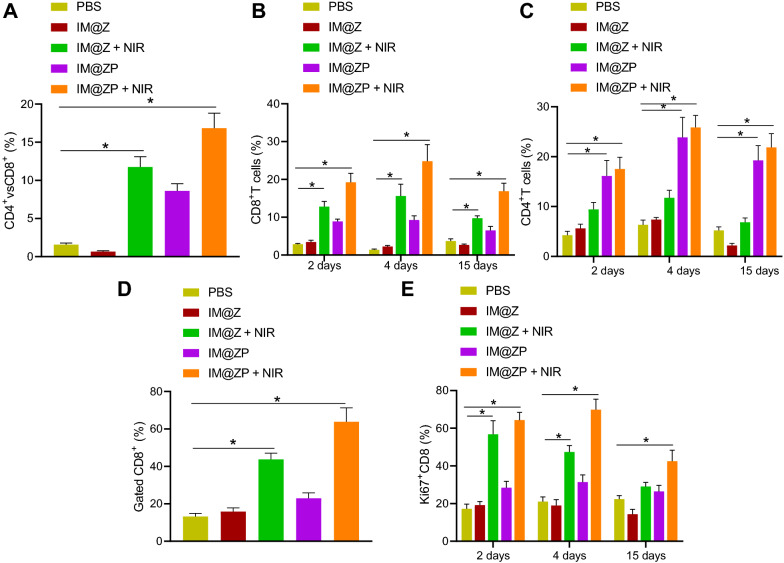
Promoted immune response by IM@ZP under NIR laser irradiation in vivo. **A** Flow cytometric analysis of CD4 +  and CD8 +  T cells in tumor tissues of mice treated with IM@Z, IM@Z  +  NIR, IM@ZP and IM@ZP  +  NIR. **B** Flow cytometric analysis of CD8  +  T cells in tumor tissues of mice treated with IM@Z, IM@Z  +  NIR, IM@ZP and IM@ZP  +  NIR. **C** Flow cytometric analysis of CD4 +  T cells in tumor tissues of mice treated with IM@Z, IM@Z  +  NIR, IM@ZP and IM@ZP  +  NIR. **D** Flow cytometric analysis of CD8  +  T cell proliferation in tumor tissues of mice treated with IM@Z, IM@Z  +  NIR, IM@ZP and IM@ZP  +  NIR. **E** Ki67-positive CD8  +  T cells in tumor tissues of mice treated with IM@Z, IM@Z  +  NIR, IM@ZP and IM@ZP  +  NIR. n  = 6 for mice following each treatment. *p  < 0.05

For better understanding of the mechanism by which IM@ZP under NIR laser irradiation inhibits tumor growth, we further analyzed its effects on tumor infiltrating CD8  +  and CD4  +  T cells in vivo. In comparison to the PBS group, the proportion of CD8  +  T cells was increased in the tumor tissues of IM@Z  +  NIR group and the IM@ZP  +  NIR group (Fig. 6A, C). Specifically, at 4 days after treatment, the proportion of CD8  +  T cells was 6.7 and 17.9 times higher in the IM@Z  +  NIR and IM@ZP  +  NIR groups than the PBS group. Additionally, 15 days after treatment, the proportion of CD8  +  T cells was still much higher in the IM@Z  +  NIR and IM@ZP  +  NIR groups than that in the PBS group. Meanwhile, the IM@ZP group and the IM@ZP  +  NIR group were found to increase CD4  +  T cells in tumor tissues than the PBS group. After 4 days of treatment, CD4  +  T cells were 3.8 and 4.1 times higher in the IM@ZP and IM@ZP  +  NIR groups than the PBS group, respectively (Fig. 6A, B). This suggested that PD-L1 silencing resulted in more CD4  +  T cell intratumor aggregation than PTT. The increase of tumor infiltrating CD8  +  and CD4  +  T cells confirmed that the combination of IM@Z  +  NIR with PD-L1 siRNA could effectively promote the anti-tumor immune response.

Flow cytometric data showed the potentiated proliferation of tumor infiltrating CD8  +  T cells in the IM@Z  +  NIR group and the IM@ZP  +  NIR group when compared with the PBS group, while the IM@ZP  +  NIR group showed the highest proliferation (Fig. 6D, E). In addition, there was an elevation in the serum levels of TNF-α and number of Natural killer (NK) cells in the IM@Z  +  NIR group and the IM@ZP  +  NIR group, when compared with the PBS group (Additional file 1: Fig. S5A, B).

Further, IM@ZP was injected in healthy mice to investigate the release of PD-L1 siRNA in vivo. In vivo pharmacokinetics experiments further confirmed that the PD-L1 siRNA circulation time was longer in the mice of the IM@ZP group, as compared with the PD-L1 siRNA group (Additional file 1: Fig. S5C).

These results further substantiated that IM@ZP enhanced photothermal immune effects through PD-L1 blockade.

### Anti-tumor and anti-metastatic effects of IM@ZP in vivo through PD-L1 blockade

**Fig. 7:**
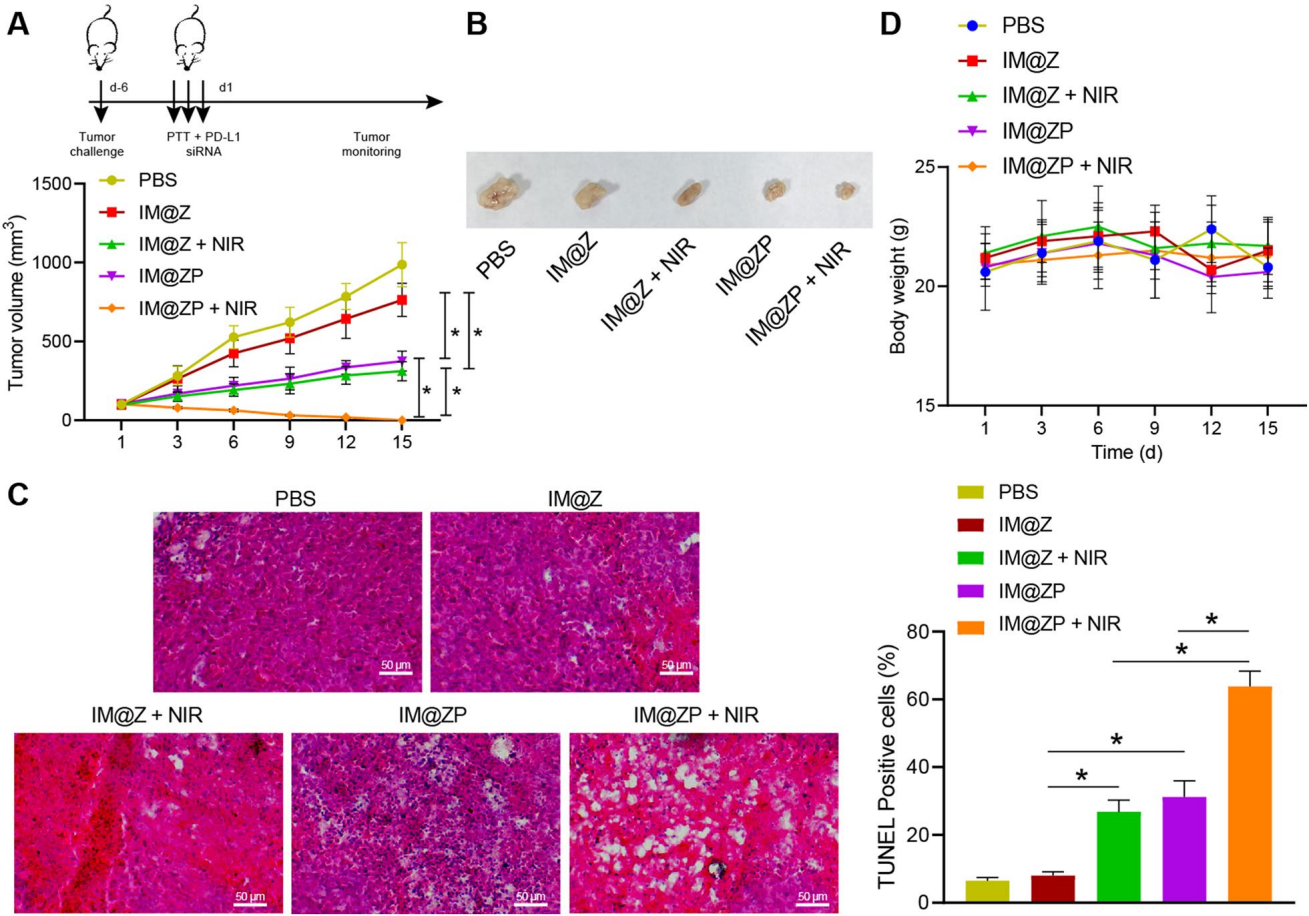
Effective anti-tumor properties of IM@ZP under NIR laser irradiation in vivo. **A** Tumor volume of mice treated with IM@Z, IM@Z  +  NIR, IM@ZP and IM@ZP  +  NIR. **B** Representative images showing xenografts in mice treated with IM@Z, IM@Z  +  NIR, IM@ZP and IM@ZP  +  NIR. **C** Cell apoptosis and pathological changes of tumor tissues in mice treated with IM@Z, IM@Z  +  NIR, IM@ZP and IM@ZP  +  NIR measured by TUNEL and HE staining assays. **D** Tumor weight of mice treated with IM@Z, IM@Z  +  NIR, IM@ZP and IM@ZP  +  NIR. n  = 6 for mice following each treatment. *p  < 0.05

We extended our mechanistic findings to determine the anti-tumor effect of IM@ZP in vivo. The results showed that, relative to PBS treatment, IM@Z treatment had no obvious effect on tumor growth. As compared to IM@Z, IM@Z  +  NIR or IM@ZP resulted in a reduction of tumor growth by about 67% and 62%, respectively. This suggested that PTT or PD-L1 silencing alone inhibited tumor growth in mice. In addition, the tumor was almost eliminated in the presence of IM@ZP  +  NIR, indicating that siRNA-mediated PD-L1 blockade could enhance IM@Z  +  NIR-mediated tumor immunotherapy (Fig. 7A, B). In addition, we also evaluated the in vivo distribution of IM@Z and IM@ZP by injecting IR792, IM@Z, and IM@ZP in tumor-bearing mice. It was found that, as compared to that of IR792, the aggregation of IM@Z and IM@ZP at tumor sites was significantly increased, corresponding to elevated fluorescence intensity of IM@Z and IM@ZP at tumor sites (Additional file 6: Fig. S6).

Further results of TUNEL and HE staining assays showed that treatment with IM@Z  +  NIR or IM@ZP could induce cell apoptosis and necrosis in tumor tissues of mice, as compared with IM@Z. This implied that PTT or PD-L1 silencing alone could augment cell apoptosis and necrosis in tumor tissues. More importantly, IM@ZP  +  NIR caused the highest percentage of apoptosis among all treatments (Fig. 7C), which was attributed to the enhanced photothermal immunotherapy effect upon downregulation of PD-L1 protein. In addition, Fig. 7D displays no obvious changes in tumor weight of mice following all treatments.

**Fig. 8:**
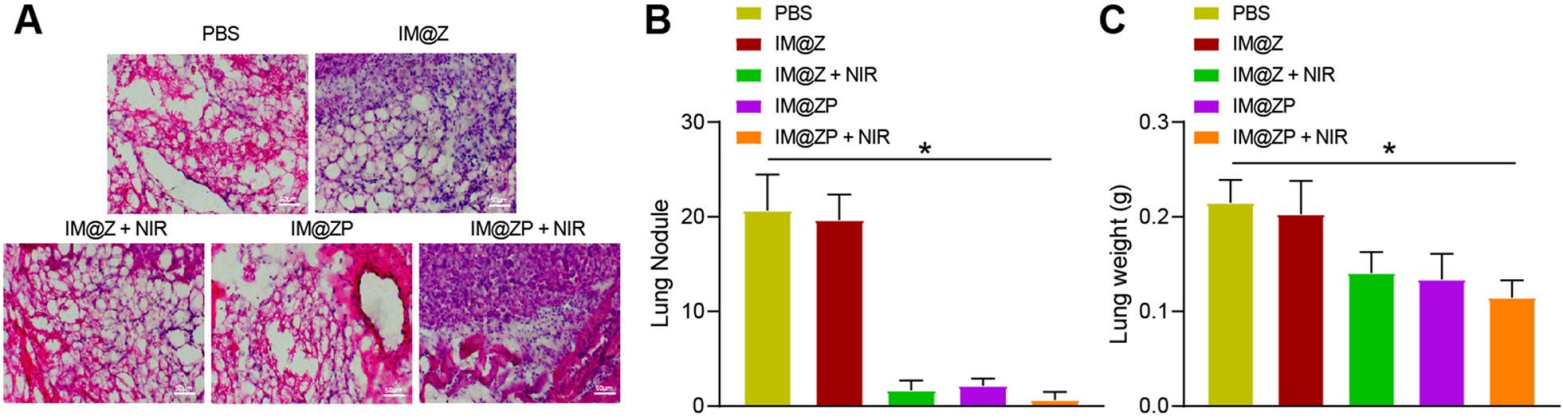
Effective anti-metastatic properties of IM@ZP under NIR laser irradiation in vivo. **A** HE staining analysis of lung metastasis of mice treated with IM@Z, IM@Z  +  NIR, IM@ZP and IM@ZP  +  NIR. **B** Lung metastatic nodules of mice treated with IM@Z, IM@Z  +  NIR, IM@ZP and IM@ZP  +  NIR. **C** Lung weight of mice treated with IM@Z, IM@Z  +  NIR, IM@ZP and IM@ZP  +  NIR. n  = 6 for mice following each treatment. *p  < 0.05

Moreover, many lung metastatic nodules were observed in most of the lung nodules in the PBS- or IM@Z-treated mice, while nodules were reduced in lung tissues of other groups. However, the least lung metastatic nodules were found in the presence of IM@ZP  +  NIR (Fig. 8A, B). The lung weight between PBS- and IM@Z-treated mice didn’t differ significantly, which was decreased in response to treatment with IM@Z  +  NIR, IM@ZP or IM@ZP  +  NIR. Among them, the mice in the presence of IM@ZP  +  NIR showed the lowest lung weight (Fig. 8C).

In conclusion, these results demonstrate that IM@ZP under NIR laser irradiation can enhance the photothermal immune effect by mediating PD-L1 blockade, which in turn inhibits breast cancer tumor growth and lung metastasis in vivo.

## Discussion

**Fig. 9:**
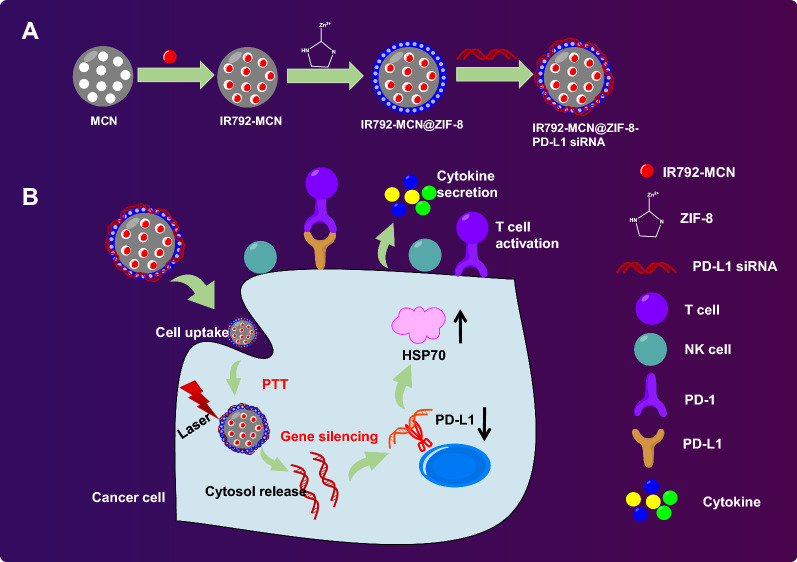
Mechanism graph. **A** IM@ZP contains two kinds of photothermal molecules (IR792 and MCN), rendering it enhanced photothermal conversion ability. **B** ZIF-8 releases siRNA into the cytoplasm in the acidic environment of tumor, showing high-efficiency characteristics to downregulate the expression of PD-L1. Under NIR laser irradiation, IM@ZP effectively kills 4T1 tumor cells, induces DC maturation and cytokine secretion to stimulate immune response

In the current study, we examined the efficacy of the IM@ZP drug delivery system in the enhanced photothermal/photodynamic/immunotherapy for TNBC and obtained the results showing that IM@ZP possessed excellent efficacy in enhancing the photothermal immunotherapy under NIR laser irradiation (Fig. 9).

We initially found that IM@ZP exhibited an efficient photothermal performance under NIR laser irradiation in vitro, ascribed to the combination of two photothermal molecules MCN and IR792. Indeed, MCN has been confirmed to have significant photothermal conversion efficiency due to its strong NIR absorption capacity [21]. Likewise, IR792 shows great NIR absorption capacity and spectroscopic and photo-physical properties [11]. Additionally, the ZIF-8-released Zn^2+^ ions can strengthen the efficacy of photothermal therapy by reducing the heat resistance of bacteria [9]. NIR-induced photothermal therapy has been a well-established promising and highly efficient option for cancer therapy, including breast cancer, whereby the elevated temperature induced by NIR laser irradiation triggers cancer cell viability inhibition [22, 23]. Consistently, the current findings indicated that the photothermal action of IM@ZP contributed to its therapeutic efficiency in breast cancer in vitro as shown by the decreased 4T1 cell viability and increased apoptosis and necrosis rates. Thus, IM@ZP may have a good application prospect for photothermal therapy.

Additionally, the present results unveiled that under 808 nm NIR laser irradiation, IM@ZP could effectively kill 4T1 cells, upregulate HSP70 expression and induce DC maturation and cytokine secretion (TNF-α and IL-6) to stimulate immune response in vitro. NIR-laser irradiation has been shown to exhibit optimized in vitro photodynamic therapy effects against the proliferation of breast cancer MCF-7 cells [24]. Besides, another study revealed that under the NIR laser irradiation, the induced phase-shifted nanoparticles can transform the photon energy into heat energy to heat up the cancer focus in a rapid manner and destroys the breast cancer tissue through photothermal therapy and photodynamic therapy [25]. HSP70 constitutes a highly-conserved member of the heat shock protein family expressed in all living organisms, and can induce anti-cancer immune response via activation of immune cells, especially antigen-presenting cells [26]. DCs are important antigen-presenting cells with the function to capture, process and present antigens to unprimed T cells, and also key players in innate and adaptive immunity, essential for the initiation of immune response [27, 28].

Further analysis in the current study uncovered the antitumor immune response and anti-metastatic effects of IM@ZP under NIR laser irradiation in vivo, which was attributed to the enhanced photothermal immunotherapy effect upon downregulation of PD-L1 protein, and the resultant promotion of tumor infiltrating CD8 +  and CD4 +  T cells and NK cells. Notably, upon 808-nm laser irradiation, a gel system combined with PD-L1 blocking could result in more effectively inhibited primary tumor growth and generate robust tumor-specific immunity against tumor recurrence and metastasis [29]. Compelling evidence has demonstrated the prognostic value of TILs, especially in TNBC where the CD4/CD8 ratio at the tumor-host interface significantly correlates to overall survival essential for both tumor size and nodal status [30]. CD8 +  T cells are essential for adaptive immunity whereby they can clear intracellular pathogens and provide permanent protection; these functions are mainly ascribed to the subpopulation of CD8 +  T cells, the cytotoxic T lymphocytes, due to their capacity to kill infected cells and to secrete cytokines including TNF-α and interferon-γ [31]. Notably, TNBC presenting high TILs may have upregulated PD-L1 expression [32], and conversely, its decrease induced by OTUB1 ablation can reduce its protein binding to the tumor cell surface, facilitate more CD8 T cell infiltration and increase the serum level of interferon-γ to enhance anti-tumor immunity in mice [33]. More importantly, PD-L1 shows an anti-tumor effect with no clear additive effects in 4T1 tumor-bearing mouse models of breast cancer [34]. Photothermal therapy has been reported to enhance cancer immunotherapy since it has immune-stimulation functions and subsequently triggers anti-tumor immune responses [35], this is very much consistent with our results, suggesting the promising usage of IM@ZP as a photothermal agent to treat TNBC.

## Conclusion

Overall, our study suggested IM@ZP nanoparticles for the enhancement of the NIR-induced photothermal cancer immunotherapy. IM@ZP contained two kinds of photothermal molecules (IR792 and MCN), rendering it enhanced photothermal conversion ability. Meanwhile, ZIF-8 could release siRNA into the cytoplasm in the acidic environment of tumor, showing high-efficiency characteristics to downregulate the expression of PD-L1. Under 808 nm NIR laser irradiation, IM@ZP could effectively kill 4T1 tumor cells, induce DC maturation and cytokine secretion to stimulate immune response. More importantly, combination with PD-L1 gene silencing can amplify the effect of photothermal immunotherapy, thus killing tumor and inhibiting its metastasis to lung. However, the specific in vivo biotechnology that underpins this process remains largely unknown, with further work required to consolidate findings of this study. Still, this novel nanoparticle system may provide an effective alternative strategy for the treatment of advanced or metastatic tumors.

## Materials and methods

### Ethics statement

Animal experiments were carried out under the approval of Animal Ethics Committee of The Affiliated Hospital of Qingdao University. Great efforts were made to minimize the number of animals used in the experiments and their discomfort.

### Synthesis and characterization of IR792-MCN

Initially, 10 mg MCN (Xianfeng Nano Company, Nanjing, China) and 5.5 mg IR792 (Sigma-Aldrich Chemical Company, St. Louis, MO, USA) were added into 1 mL water, and subjected to ultrasound treatment for 2 min. All operations were conducted in the dark. Next, the mixture was stirred overnight at room temperature and centrifuged at 12,000 rpm for 10 min to remove the free IR792 to obtain IR792-MCN. The encapsulation efficiency was subsequently calculated using UV–VIS spectrophotometer (DU800, Beckman Coulter Inc., USA). XPS was then performed using an Axis Ultra spectrometer (Kratos, Manchester, UK) to determine the nanocrystal surface.

### Synthesis of MCN@ZIF-8 and IM@Z

To confirm the successfully synthesis of ZIF-8 in situ on the surface of MCN, 10 mg of MCN and IR792-MCN, and 100 mg of zinc nitrate were dissolved in 5 mL of methanol and treated with ultrasound for 2 min. Thereafter, 2-methylimidazole (Sigma-Aldrich, 165 mg 2-methylimidazole was dissolved in 5 mL methanol solution) was added to the mixture, and incubated in a water bath at 25 °C, followed by magnetic stirring for 10 min. Finally, the mixture was centrifuged and washed thrice with PBS to obtain pure MCN@ZIF-8 and IM@Z before freezing and drying for further use. Morphological evaluation and element analysis of the prepared MCN@ZIF-8 and IM@Z samples were performed using a TEM (HT7700, Hitachi Chemical, Tokyo, Japan). The absorbance curve of IR792 and IM@Z was determined by UV–VIS spectrophotometer (DU800, Beckman).

### Synthesis of IM@ZP

PD-L1 siRNA was dissolved in diethypyrocarbonate (DEPC; 50 µM; Sigma-Aldrich). Then, 1 mg of IM@Z (Na) was dispersed into 1 mL of PD-L1 siRNA (4 μM) and stirred for 30 min at room temperature. The FAM-labeled PD-L1 siRNA (siRNA-FAM; Shanghai GenePharma Co., Ltd., Shanghai, China) was adsorbed to the surface of IM@Z by electrostatic interaction. Afterwards, the free PD-L1 siRNA was removed by centrifugation at 10,000 rpm for 10 min, and the IM@ZP pellet was collected and resuspended in 100 µL DEPC (10 mg/mL). For preliminary verification of IM@Z for PD-L1 siRNA loading, Malvern Zetasizer Nano ZS (Malvern Instruments, Worcestershire, UK) was used to measure the potential change of IM@Z with siRNA binding. Furthermore, gel electrophoresis experiments were carried out to study the ability of different mass ratios (0.5, 1, 2, 5, 10, 15 and 20) of IM@Z for siRNA adsorption. In this experiment, 2 µL siRNA and 8 µL IM@Z were added to each group with a final volume of 100 µL. The mixture was incubated at 37 °C for 30 min, and centrifuged whereupon the remaining siRNA in the supernatant was collected. The adsorption capacity was evaluated by electrophoresis. Pure siRNA was used as a negative control.

### Release of siRNA and its serum stability

To achieve high transfection efficiency, the release of siRNA from the IM@ZP was investigated. siRNA-FAM was used for this experiment. Briefly, 1 mg/mL IM@ZP was incubated with PBS at different pH values (5.0, 6.0 and 7.4) at 37 °C for 0–8 h, followed by centrifugation. Next, the content of siRNA-FAM in the supernatant was quantified by fluorescence spectrometry, and the release rate of siRNA was thus calculated.

Subsequently, its stability in serum was analyzed. Initially, 10 mg/mL IM@ZP (20 µL) was put in 180 µL fetal bovine serum (FBS; Hyclone Laboratories, Logan, UT, USA) and then incubated at 37 °C for 1–4 h. IM@ZP was collected by centrifugation and put in PBS (pH 5.5) to dissolve ZIF-8 and release siRNA. Next, 4% (w/v) agarose gel electrophoresis was used to assess the stability. Pure siRNA of an equal concentration with IM@ZP served as the control.

### Photothermal experiments in vitro

The temperature change of MCN@ZIF-8-PD-L1 siRNA and IM@ZP at different concentrations (5, 10, 25, 50, 100 and 200 µg/mL) was measured in real time by thermocouple probe under 808 nm laser irradiation at a power of 1 W/cm^2^ for 300 s. Meanwhile, the temperature change of 50 μg/mL MCN@ZIF-8-PD-L1 siRNA and IM@ZP under 808 nm laser irradiation at different powers (from 0.5 to 1.5 W/cm^2^) was measured.

### Cell culture

Mouse breast cancer 4T1 cell line (RRID: CVCL_0125) and 4T1 cell line expressing green fluorescent protein 4T1/GFP/FlucII (RRID: CVCL XG69) were purchased from the Cell Bank of Chinese Academy of Sciences (Shanghai, China) and Tongpai (Shanghai) Biotechnology Co., Ltd. (Shanghai, China) respectively. The cells were cultured in Roswell Park Memorial Institute (RPMI) 1640 medium containing 10% FBS, 100 U/mL penicillin (Hyclone) and 100 μg/mL streptomycin (Hyclone) in a 5% CO_2_ incubator at 37 °C. The cells were seeded in confocal plates, 96-well plates, 24-well plates or 6-well plates.

### Cell uptake and transfection

FAM-modified PD-L1 siRNA and IR792 fluorescence signal were applied for the evaluation of IM@ZP uptake in 4T1 cells. Briefly, 4T1 cells were seeded into 24-well plates at a density of 5 × 10^4^ cells/well and cultured for 24 h. Then the cells were treated with 100 µg mL^−1^ IM@ZP (the mass ratio of vector to siRNA was 80:1) for 1, 2, 4 and 6 h and washed with PBS for several times. Thereafter, the cells were trypsinized, whereupon the uptake of IM@ZP was determined by a Calibur flow cytometer (BD Biosciences, San Jose, CA, USA) following incubation at different time points. IM@ZP localization in 4T1 cells was subsequently observed. In short, the 4T1 cells were seeded on the confocal plate and intubated with 100 µg mL^−1^ IM@ZP for 1, 2, 4 and 6 h. Following PBS washing, the cells were fixed with 4% paraformaldehyde (200 μL) at 4 °C for 10 min. The cells were washed twice with PBS, and the nuclei were stained with 4′,6-diamidino-2-phenylindole (DAPI; 1 μg/mL in PBS; Yeasen Biotechnology, Shanghai, China) for 15 min. Finally, the uptake was observed under a CLSM (TCSNT1, Leica, microsystems, Heidelberg, Germany) (FAM: Ex  = 490 nm, Em  = 520 nm).

Next, the release of siRNA in cells was analyzed using FAM-modified PD-L1 siRNA and LysoTracker Red fluorescence signal. 4T1 cells were seeded into the laser confocal dish at a density of 5 × 10^4^ cells/well for 24 h of culture, and then treated with IM@ZP for 0, 1, 2 and 4 h. Afterwards, DAPI and LysoTracker Red were used to stain the nuclei and lysosomes, respectively before observation under a CLSM.

### Transfection efficiency of siRNA in vitro

To achieve efficient siRNA transfection, the mass ratio of IM@Z and siRNA was optimized and meanwhile, a laser confocal microscope and flow cytometer were used to analyze the transfection efficiency of GFP-siRNA (GFP sense: 5′-GGCUACGUCCAGGAGCGCACC-3′), with NC-siRNA (NC sense: 5′-CGGUGAGCCAGGCGUGCAAUU-3) used as a parallel control. In brief, 4T1-GFP cells were seeded into 24-well culture plates at a density of 5 × 10^4^ cells/well and cultured for 24 h. IM@Z at different mass ratios (20:1, 40:1, 60:1, 80:1 and 100:1) bound to siRNA (80 nM) after which the sample was incubated with cells for 12 h, and the supernatant medium was discarded. Following three washes with PBS, the medium was renewed and the cells continued to culture for 36 h, with the expression of GFP examined. PBS was used as a control to calculate the transfection efficiency.

### Western blot analysis

According to the above experiments, the best mass ratio of 80:1 was used to synthesize three kinds of IM@ZP (the concentration of siRNA was 40, 80 and 160 nM) and Western blot analysis was used to evaluate the feasibility of downregulating PD-L1 protein. 4T1 cells were cultured in a 6-well culture plate and added with IM@ZP for 12 h of incubation. The medium was then renewed and the cells were incubated for another 36 h, with the expression of PD-L1 protein (1: 1000, ab213480, Abcam, Cambridge, UK) in cells analyzed and semi-quantified by the Image J software (National Institutes of Health, Bethesda, Maryland, USA). To preliminarily elucidate the tumor immune mechanism of IM@ZP in vitro [36], the expression of HSP70 (1: 1000, ab2787, Abcam) was evaluated according to the aforementioned procedure.

### Cytotoxic effect evaluation in vitro

CCK-8 assay (Dojindo Molecular Technologies, Japan) was performed to analyze the cytotoxic effect of IM@ZP on 4T1 cells. Briefly, 4T1 cells were seeded into 96-well culture plates at a density of 4 × 10^3^ cells/well and cultured for 24 h, followed by treatment with an equal volume of PBS, IM@Z and IM@ZP (100 µg/mL) for 12 h. The experimental group with light exposure was irradiated with 808 nm laser (1 W/cm^2^) for 10 min. The fresh medium was replaced and the cells were incubated for another 12 h, after which cell viability was determined by CCK-8 assay. The optical density (OD) value of untreated cells was set as a blank, and the cell activity was calculated using the following formula: cell activity (%)  =  [OD value (sample) − OD value (blank)]/[OD value (control) − OD value (blank)].

Subsequently, fluorescence microscope and flow cytometer were used to detect the staining and apoptosis of living/dead cells. Briefly, 4T1 cells were seeded into 6-well plates at a density of 2 × 10^5^ cells/well and cultured for 24 h, followed by treatment with PBS, IM@Z and IM@ZP as previously. The treatment is consistent with the previous steps. Part of the cells was stained with 2 μM Calein AM and 50 μg/mL propidium iodide (PI) for 10 min and the living/dead cells were observed under a fluorescence microscope (Leica). Another part of the cells were trypsinized, transferred to a centrifuge tube, washed twice with PBS, and centrifuged at 1000*g* for 5 min, with the supernatant discarded. Thereafter, the cells were collected and resuspended in 100 μL binding buffer. Annexin V-phycoerythrin (PE; 5 μL; Biolegend, San Diego, CA, USA) and 7-aminoactinomycin D (7-ADD; 10 μL; Biolegend) were added to the cells and incubated at room temperature in the dark for 15 min. Finally, the cells were added with 400 μL binding buffer and mixed before apoptosis analysis using a Calibur flow cytometer (BD Bioscience).

### Induction of dendritic cell (DC) maturation in vitro

DCs were isolated from the bone marrow of 8-week-old female C57BL/6 mice (purchased from Hunan SJA Laboratory Animal Co., Ltd., Hunan, China). In short, mouse bone marrow cells were obtained by washing tibia and femur with PBS containing 2% FBS. Subsequently, the cells were collected and cultured in X-vivo 15 medium (Lonza, Switzerland) containing GM-CSF (20 ng/mL) and IL-4 (10 ng/mL) for 5 days to obtain immature DCs. On the 6th day, immature DCs were co-incubated with 4T1 cells treated with PBS, IM@Z, IM@Z  +  NIR, IM@ZP and IM@ZP  +  NIR (100 µg/mL). After 24 h, DCs were collected and probed with anti-mouse CD11c-PE antibody (eBioscience, San Diego, CA), anti-mouse CD86-APC antibody (eBioscience) and anti-mouse CD80-FITC antibody (eBioscience), followed by analysis using flow cytometer. Meanwhile, levels of TNF-α and IL-6 were determined in the supernatant of DCs using their separate ELISA kits (InvivoGen, San Diego, CA, USA).

### Xenograft tumor in mice

Female BALB/c mice (5–6 weeks) purchased from Hunan SJA Laboratory Animal Co., Ltd. (Hunan, China) were raised in specific pathogen-free animal laboratory with controlled temperature (22–25 °C), relative humidity (60–65%), and illumination (12 h light–dark cycle). With free access to food, the mice were acclimated for one week before experiments. Healthy status of the mice was observed before the experiment.

For subcutaneous tumor model construction, 100 μL PBS containing 1 × 10^6^ 4T1 cells was subcutaneously injected into the left shoulder side of the mice. For the lung metastasis model, 1 × 10^6^ 4T1 cells were subcutaneously injected into the left side of the mice, and 2 × 10^5^ 4T1 cells were injected into the mice via tail vein on the 6th day as circulating tumor cells (CTCs). Tumor volume was calculated as follows: tumor volume (mm^3^)  =  length  ×  width^2^/2.

### Antitumor effects in vivo

When the tumor volume reached approximately 100 mm^3^, 100 μL normal saline, IM@Z and IM@ZP were injected to mice via tail vein respectively (the dose of siRNA was 0.31 mg/kg). In the light group, 808 nm NIR laser with power of 1 W/cm^2^ was used to irradiate the tumor site for 10 min. For this experiment, mice were divided into five groups (n  = 6): PBS, IM@Z, IM@Z  +  NIR, IM@ZP and IM@ZP  +  NIR. Tumor volume and weight of mice were monitored daily. At the end of anti-tumor treatment, tumor and major organs were collected and subjected to hematoxylin–eosin (HE) and terminal deoxynucleotidyl transferase-mediated dUTP-biotin nick end labeling (TUNEL) staining.

Furthermore, the anti-tumor and anti-metastatic actions of IM@ZP were dissected out. Briefly, one day after the lung metastasis model was established by injection of 4T1 cells into the mouse tail vein, the mice were treated with PBS, IM@Z, IM@Z  +  NIR, IM@ZP and IM@ZP  +  NIR. On the 18 day after treatment, the mice were euthanized. The lung tissues of mice were removed, photographed and weighed, after which the number of metastatic nodules was calculated, and the sections were stained with HE.

### Flow cytometry for immune cells in vivo

The effect of IM@ZP on the content and proliferation of CD4  +  and CD8  +  immune cells in tumor was elucidated. On the 2, 4 and 15 day after treatment, the tumor was collected from mice and digested with 1500 U/mL collagenase (Sigma-Aldrich), 1000 U/mL hyaluronidase (Sigma-Aldrich) and DNase (Sigma-Aldrich) for 30 min at 37 °C. Tumor-infiltrating lymphocytes (TILs) were separated by filtration through a nylon mesh filter and then enriched. After washing with PBS containing 1% FBS, TILs were incubated with anti-CD3-PerCP Cy5.5, anti-CD8a-PE10 and anti-CD4-FITC antibodies (eBioscience) at 4 °C for 30 min. The content of immune cells was then detected by a flow cytometer. For Ki67 analysis, TILs were fixed, dissolved in FoxP3 buffer, and immunostained with anti-Ki67-FITC (eBioscience). The proliferation of TILs was analyzed by a flow cytometer. For cytokine detection, serum samples were collected and analyzed with a TNF-α ELISA kit. NK cells stained with anti-CD3-PerCP Cy5.5 (eBioscience) and anti-CD49b-FITC (eBioscience) antibodies were analyzed using a flow cytometer.

### TUNEL assay

On the 10th day after administration, one mouse was collected from each group and the subcutaneous tumor was stripped away from the surrounding tissues. The tumor tissues were then fixed in a container containing 4% paraformaldehyde for 48 h, dehydrated, immersed in 15% (w/w) sucrose solution for 24 h and in 30% sucrose solution for another 24 h. Next, the tissues were embedded with Tissue-Tek O.C.T. Compound, and cut into 8-μm-thick sections, which were adsorbed on the cationic glass slide. The sections were permeabilized in the freshly prepared 1% Triton X-100 solution at room temperature for 3–5 min and rinsed thrice with PBS (5 min per rinse). Next, each sample was added with 50 μL TDT enzyme reaction solution (each sample size: 45 μL equilibrium buffer  + 1 μL biotin-11-dUTP) and reacted in the dark at 37 °C for 60 min. Afterwards, each sample was reacted with 50 μL labeling buffer (45 μL labeling buffer  +  5 μL streptavidin-FITC) for 30 min at 37 °C under dark conditions. Following PBS washing, the sample was subjected to DAPI (10 μg/mL, 50 μL) staining for 15 min and observed under a fluorescence microscope. FITC: Ex  =  488 nm; DAPI: Ex  =  405 nm.

### In vivo pharmacokinetics experiments

Healthy BALB/c mice were randomly divided into two groups: PD-L1 siRNA and IM@ZP (6 mice per group). The PD-L1 siRNA group was taken as a control group to investigate the in vivo kinetic characteristics of EXO-PTX. Before intravenous administration into tails, mice were fasted 12 h and allowed with free access to drinking water. After administration, blood (about 100 μL) was collected from the jugular sinus of mice at 0.25, 0.5, 1, 2, 4, 8, 12 and 24 h, respectively. Blood was immediately transferred to the ice-cold heparin-containing centrifuge tube, followed by centrifugation at 3000 rpm for 10 min. The supernatant was harvested and stored at − 80 °C. The in vivo release of PD-L1 siRNA was detected by qPCR and the pharmacological parameters were calculated.

### Small animal in vivo imaging

Before administration, tumor-bearing mice were randomly divided into three groups: IR792 IM@Z and IM@ZP (6 mice per group). Mice were injected (100 μL) into caudal vein with IR792, IM@Z and IM@ZP. In vivo distribution of IR792, IM@Z and IM@ZP in mice was observed by small animal in vivo imaging at 0, 1 and 2 h after injection. The mice were euthanized 24 h after injection. The main organs (heart, liver, spleen, lung, kidney) and tumors were removed. The fluorescence distribution of each organ was statistically analyzed.

### Statistical analysis

Statistical analyses were performed using the GraphPad Prism software (GraphPad Software, La Jolla, CA, USA). Data were expressed as mean  ±  standard deviation. Data between two groups were compared using independent sample *t* test. Data among multiple groups were compared by one-way analysis of variance (ANOVA), followed by a Tukey multiple comparisons post-test. Repeated measures ANOVA with Bonferroni post hoc test was applied for the comparison of data at different time points. A value of *p*  < 0.05 was indicative of statistical significance. Each experiment was repeated for three times independently.

## Supplementary Information

**Additional file 1: Figure S1.** The mechanism of the formation of silica nanoparticles by hydrolysis of tetraethylorthosilicate (TEOS).

**Additional file 2: Figure S2.** MCN absorption and photothermal conversion efficiency under NIR irradiation. **A** The ultraviolet absorption of MCN was measured by UV/VIS spectrophotometer. **B** Temperature curves of MCN, IR792-MCN and MCN@ZIF-8-PD-L1 under 1 W/cm^2^ NIR laser irradiation for 3 min.

**Additional file 3: Figure S3.** Localization of IM@ZP (red) in 4T1 cells. CLSM images of IM@ZP (100 μg/mL) incubated with 4T1 cells for 6 h, with DAPI staining for nuclei.

**Additional file 4: Figure S4.** Western blots (**A**) and quantitative analysis (**B**) of HSP70 protein in 4T1 cells treated with IM@Z + NIR, IM@ZP and IM@ZP + NIR. *p < 0.05.

**Additional file 5: Figure S5.** Serum levels of TNF-α and number of NK cells in the in vivo immune stimulation of IM@ZP under NIR laser irradiation. **A** Levels of TNF-α in the serum of mice treated with IM@Z + NIR, IM@ZP and IM@ZP + NIR measured by ELISA. **B** Number of NK cells in tumor tissues of mice treated with IM@Z + NIR, IM@ZP and IM@ZP + NIR. **C** In vivo pharmacokinetic curves of IM@ZP and PD-L1 siRNA in mice. n = 6 for mice following each treatment. *p < 0.05.

**Additional file 6: Figure S6.** In vivo biological distribution of IM@Z and IM@ZP. Fluorescence distribution of each organ of mice treated with IR792, IM@Z and IM@ZP. n = 6 for mice following each treatment. *p < 0.05.

## Data Availability

The datasets generated/analysed during the current study are available.
